# Association of Mercury Exposure and Maternal Sociodemographics on Birth Outcomes of Indigenous and Tribal Women in Suriname

**DOI:** 10.3390/ijerph18126370

**Published:** 2021-06-12

**Authors:** Gaitree K. Baldewsingh, Ashna D. Hindori-Mohangoo, Edward D. van Eer, Hannah H. Covert, Arti Shankar, Jeffrey K. Wickliffe, Lizheng Shi, Maureen Y. Lichtveld, Wilco C. W. R. Zijlmans

**Affiliations:** 1 Medical Mission Primary Health Care Suriname, Paramaribo, Suriname; evaneer@gmail.com; 2 Faculty of Medical Sciences, Anton de Kom University of Suriname, Paramaribo, Suriname; wilco.zijlmans@uvs.edu; 3 Tulane University School of Public Health and Tropical Medicine, New Orleans, LA 70112, USA; ashna.mohangoo@perisur.org (A.D.H.-M.); hcovert@tulane.edu (H.H.C.); sarti@tulane.edu (A.S.); jkwickli@uab.edu (J.K.W.); lshi1@tulane.edu (L.S.); mlichtve@pitt.edu (M.Y.L.); 4 Foundation for Perinatal Interventions and Research in Suriname (Perisur), Paramaribo, Suriname; 5 Department of Environmental Health Sciences, School of Public Health, University of Alabama at Birmingham, Birmingham, AL 35294, USA; 6 Graduate School of Public Health, University of Pittsburgh, Pittsburgh, PA 15261, USA

**Keywords:** adverse birth outcome, preterm birth, ethnicity, indigenous, tribal, mercury exposure, Suriname

## Abstract

Information regarding adverse birth outcomes (ABO) of Indigenous and Tribal women living in the remote tropical rainforest of Suriname, where mercury (Hg) use is abundant in artisanal gold mining, is not available. In the context of a health system analysis, we examined the association between Hg exposure, maternal sociodemographics on the ABO of Indigenous and Tribal women living in Suriname’s interior and its capital, Paramaribo. ABO were determined in pregnant women enrolled from December 2016 to July 2019 in the Caribbean Consortium for Environmental and Occupational Health prospective environmental epidemiologic cohort study. Associations were explored using Pearson’s χ^2^-test and the Mann–Whitney U-test. Among 351 singleton participants, 32% were Indigenous, residing mainly in the interior (86.8%), and 23.1% had ABO. Indigenous participants had higher rates of ABO (29.8% vs. 19.8%) and preterm birth (PTB) (21.2% vs. 12.4%), higher Hg levels, delivered at a younger age, were less educated, and had lower household income compared to Tribal participants. Multivariate logistic regression models revealed that Indigenous participants had higher odds of ABO (OR = 3.60; 95% CI 1.70–7.63) and PTB (OR = 3.43; 95% CI 1.48–7.96) compared with Tribal participants, independent of Hg exposure and age at delivery. These results highlight the importance of effective risk reduction measures in support of Indigenous mothers, families, and communities.

## 1. Introduction

Ethnic disparities in adverse birth outcomes (ABO) in Suriname are not well documented. A recent nationwide birth registry revealed substantial inequities by ethnicity with Tribal women experiencing the highest risk of ABO (maternal mortality, stillbirth, increased preterm birth, low Apgar score) [1]. ABO such as preterm birth (PTB) and low birthweight (LBW) have serious health consequences across the course of life [2]. A number of maternal sociodemographic disparities are associated with ABO, such as maternal education, maternal occupation, and household income [3]. Though notable progress has been made in reducing ABO in Suriname, the gap between infants born to women with unfavorable sociodemographic characteristics continues to demand practical and culturally appropriate public health intervention programs [1].

Prematurity is an important ABO and is associated with severe morbidity and increased mortality; for example, infants born between 33 and 37 weeks of pregnancy have a higher risk of airway disorders [4,5]. Maternal determinants such as smoking, low body mass index, previous PTB, social deprivation, levels of education, unemployment, single motherhood, and maternal age of <20 years or >35 years are all associated with a higher risk of prematurity [6]. Stillbirth, another important ABO, accounts for approximately 7% of the global burden of disease and is often associated with maternal morbidity and long-lasting psychosocial distress for mothers [7]. A nationwide study of perinatal outcomes in Suriname in 2016 and 2017 reported the country to have the second-highest stillbirth rate in Latin America and the Caribbean (14.8 per 1000) [8].

For pregnant women enrolled in the Caribbean Consortium for Environmental and Occupational Health (CCREOH) prospective environmental epidemiologic cohort study, Hg exposure was significantly associated with PTB, perceived stress was significantly associated with a low Apgar score, while depression was not associated with any birth outcomes [9]. ABO such as LBW and low Apgar (<7 at 5 min) are also associated with infant mortality and infant and childhood morbidity [10,11]. In Suriname, the prevalence of LBW was recently reported to be 15%, which is higher than the overall average in most Latin American and Caribbean countries (10%), but lower than Haiti (23%) and Guyana (16%). For low Apgar, the prevalence was 3.9%, which is higher than Brazil (0.4%) [1,12,13].

Suriname is a middle-income country in northeast South America with an estimated, multi-ethnic population of 575,991 [14]. Most of its inhabitants reside in the urban areas and the remainder in rural areas, such that 10% of the population lives in the remote tropical rainforest interior. Of the interior population, 83% are Tribal (individuals of African descent) and 17% are Indigenous (Amerindians). Data on differences in ABO in these two ethnic subpopulations residing in the interior and in the capital, Paramaribo, are not available. A recent study observed 22% ABO in the total interior population, but no comparison was made with the capital [15]. Another study reported stillbirth to be highest in the Tribal population living in the capital [1].

Pregnant women living in the remote interior of Suriname are known to experience relatively high exposures to mercury (Hg) [16,17] and have a significantly higher rate of ABO, especially PTB and LBW [15]. Mercury has been listed by the World Health Organization (WHO) as one of the most toxic agents for public health concerns. Exposure to Hg has been associated with significant morbidity and mortality in exposed adults and children [18]. Hg crosses the placenta and evidence suggests that the developing fetus is sensitive to its neurotoxic effects [19]. Hg exposure in pregnancy has been associated with both pregnancy complications and neurodevelopmental delay in infants [19,20]. In Suriname, Hg is widely used in the interior, primarily in artisanal small-scale gold mining activities [21]. Hg is also used in gold mining in other Latin American and Caribbean countries such as Guyana, Peru, and Colombia [22]. The hazardous form to human health is methylmercury (MeHg), which accumulates in fish. People living in the interior highly depend on locally caught fish for their main protein source [21]. Recognizing the adverse effects of Hg exposure to health and environment, Suriname committed to the Minamata Convention on Hg in March 2018 [23].

The ongoing CCREOH study measures prenatal exposure to several heavy metals and elements, pesticides, and non-chemical stressors and their association with adverse maternal and pediatric health outcomes. Despite the high levels of Hg exposure and the potentially high incidence of LBW and PTB in Suriname’s remote interior, no studies have compared the incidence of ABO in the interior with ABO in the urban capital of Suriname, which has more favorable sociodemographic conditions and less Hg exposure [16,24]. This study aimed to examine the association between Hg exposure, social determinants (maternal age, parity, education level, household income), and ethnicity on birth outcomes of Indigenous and Tribal women in the rural interior and the capital of Suriname. We hypothesized that living in a remote area with less favorable sociodemographic conditions and higher exposure to Hg is associated with a higher risk for ABO (stillbirth, PTB, LBW, low Apgar score).

## 2. Materials and Methods

### 2.1. Study Population

The study population is composed of a sub-cohort of pregnant women living in the capital, Paramaribo, and in the remote tropical rainforest interior of Suriname as part of the CCREOH prospective environmental epidemiologic cohort study, which addresses the impact of chemical and non-chemical environmental exposures in mother/child dyads. The full study details are described elsewhere [17]. Briefly, 1200 women aged 16–45 years were recruited from December 2016 through July 2019. Data were collected using pretested, culturally appropriate questionnaires during pregnancy by trained recruiters. The questionnaires assessed demographic and lifestyle factors that may contribute to Hg exposure such as dietary factors and occupational exposures. Birth outcomes and antenatal care (ANC) measures were collected from the labor and delivery medical records where the baby was born. ANC measures included were as follows: monitoring of weight, blood pressure and blood sugar, deworming, toxoid immunization, laboratory testing for malaria, HIV, hepatitis B and syphilis, blood and urine testing, and iron and folic acid supplementation. Women aged 16 years and older with a singleton pregnancy who had registered at 1 of the 4 hospitals in the urban capital Paramaribo and 1 of the 15 randomly selected health centers in the interior were eligible to enroll in the study. This study focused on a health system analysis assessing ABO in urban and rural regions of the country and was restricted to sociodemographic determinants. Given this focus, we did not include the biological factors in this study. Specifically, we explored the role of differences in health care service systems in ABOs in women exposed to Hg, taking into account the unique sociodemographic characteristics between those living in the interior compared to participants living in the capital, Paramaribo, which is served by a different health system.

**Figure 1:**
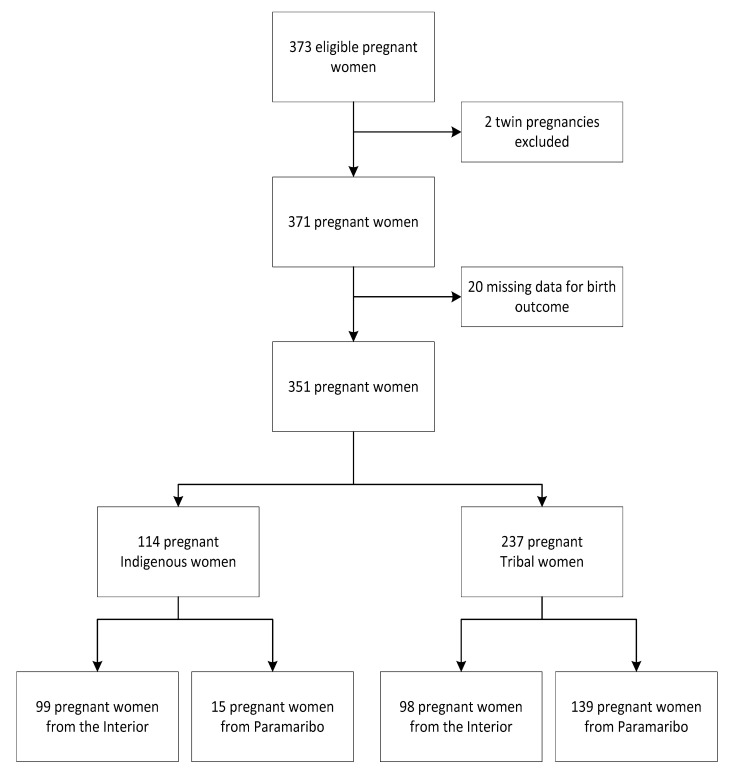
Enrollment flowchart of the study population.

In total, 975 participants were recruited in Paramaribo (*n* = 768) and in the interior (*n* = 207). Our current study population was restricted to the Indigenous and Tribal participants (*n* = 373) in both Paramaribo and the interior (Figure 1). Two recruited participants were excluded because of multiple gestation (*n* = 371). We distinguished Indigenous from Tribal participants because pregnant women from different cultural backgrounds may have different views and beliefs about pregnancy and follow different lifestyles. These two communities live in distinct villages and have little interaction. Data on the ABO of 20 Tribal participants were missing (19 from Paramaribo and 1 from the interior) and were therefore excluded from the analysis (*n* = 351). Written informed consent was obtained from all participants, while assent was obtained for participants aged 16–17 years. Human subject protection approval was granted by the Institutional Review Board of the Government of Suriname (VG 023-14) and the Tulane University Institutional Review Board (839093).

### 2.2. Mercury Levels

Hair samples of at least 1.5 g from all pregnant women were collected according to protocol, by cutting strands close to the scalp from the occipital region of the head, storing them at room temperature in a climate-controlled room, and sending them to the Environmental Research Center laboratory of the Anton de Kom University of Suriname for total Hg analysis using cold vapor atomic absorption spectrometry (CVAAS). Hair samples were rinsed with ethanol. Samples (0.5 g dry weight) were digested in 250 mL containers using 2.5 mL of both ultrapure sulfuric acid (H_2_SO_4_) and nitric acid (HNO_3_) for 12 h at room temperature, and then heated to 75 °C for 1 h; 100 mL of ultrapure deionized water was added to the digested solution with 15 mL of 5% potassium permanganate (KMnO_4_). Samples were then placed in a 95 °C water bath for 2 h and then allowed to cool. After cooling, 6 mL sodium chloride-hydroxylamine and 5 mL tin(II) chloride (SnCl_2_) were added to the hair samples. Mercury measurements were performed using a Bacharach mercury analyzer following cold vapor atomic absorption techniques [24,25]. The US Environmental Protection Agency (USEPA) reference dose for methylmercury is 0.1 µg/kg body weight/day, which corresponds to a hair concentration of 1.1 µg/g [26]. The USEPA therefore uses an action level of 1.1 µg/g for decisions regarding interventions or follow-up. For participants who had missing hair samples, existing blood Hg levels were converted to hair Hg levels when possible (*n* = 38; 12.5% of Hg levels). The USEPA uses a hair/blood Hg ratio and conversion factor of 250, which was used in this study [25].

### 2.3. Outcome Variables

The main dependent outcomes were ABO (yes vs. no), which were defined by the presence of one or more of the following birth outcomes: (i) stillbirth (defined as a fetus born with no signs of life from 22 completed weeks of gestation or a birthweight of 500+ g (yes vs. no)); (ii) PTB (defined as a birth between 22 + 0 and 36 + 6 weeks of gestation (yes vs. no)); (iii) LBW (defined as a birthweight less than 2500 g, regardless of gestational age at birth (yes vs. no)); and (iv) low Apgar score at 5 minutes (defined as a score less than seven at 5 minutes after birth (yes vs. no)).

### 2.4. Covariates

Ethnic and geographic backgrounds were dichotomized into ‘Indigenous vs. Tribal’ ethnicity and ‘Interior vs. Paramaribo’, respectively. Hg levels were first categorized into tertiles based on their distribution: low (<1.24 µg/g), medium (1.24–3.28 µg/g), and high (>3.28 µg/g) exposure. Because of small numbers for Indigenous in the lowest tertile (*n* = 8), low and medium exposure subgroups were combined versus the high exposure subgroup. Maternal demographics were analyzed as categorical variables: age at birth (16–19 vs. 20–34 vs. 35+ years), parity (no vs. one or more previous live births), educational level (primary or no vs. secondary or tertiary), and household income (<1500 vs. 1500+ Surinamese dollars (1 SRD = 0.13 USD).

### 2.5. Statistical Analyses

Descriptive statistics were calculated for the study population, stratified for the ethnic and geographic background of the participants, and presented as the median with interquartile range (IQR) for continuous variables and numbers/proportions for categorical variables. Distributions of continuous variables were tested for normality using the Shapiro–Wilk test; differences by ethnic and geographic subgroups were tested with the Mann–Whitney U-test. A two-sample test was used to test differences in proportions of ABO between the ethnic and geographic subgroups. Associations between categorical variables were tested using the Pearson’s χ^2^-test. Bivariate and multivariate logistic regression models were used to explore whether the ethnic and geographic background of the participant was associated with ABO independent of prenatal Hg exposure and non-ethnic, non-geographic maternal demographics. Multicollinearity between maternal demographics was explored using the variance inflation factor. For the final multivariate model, non-significant covariates were removed stepwise until all remaining variables had a *p*-value of 0.05 or less. All analyses were conducted using IBM SPSS Statistics for Windows version 27.0 (IBM Corp. Released 2020, Armonk, NY).

## 3. Results

Table 1 shows the distribution of the study population stratified for 114 Indigenous and 237 Tribal participants. Compared with Tribal participants, Indigenous participants had higher levels of hair Hg (median 7.9 vs. 1.4 µg/g; *p* < 0.001) and delivered at younger ages (median 25.1 vs. 29.1 years; *p* = 0.001). In addition, Indigenous women were more often less educated (79.6% vs. 45.3%, with either a primary educational level or not educated; *p* < 0.001) and had a lower household income (83.9% vs. 53.9%, with an income <1500 SRD; *p* < 0.001). The timing of the first ANC visit was later in pregnancy for Indigenous participants (median 15 vs. 12 weeks; *p* = 0.017). The observed differences in Hg exposure and maternal demographics between Indigenous and Tribal participants were not apparent for Paramaribo (see Supplementary Table S1); for the interior, the association between subgroups of Hg exposure/maternal age and Indigenous/Tribal ethnic background was significant.

Table 2 shows the distribution of the study population stratified for geographic region with 197 participants from the interior and 154 from Paramaribo. The interior participants were more often Indigenous (50.3% vs. 9.7%; *p* < 0.001). Compared with Paramaribo, interior participants had higher levels of hair Hg (median 3.6 vs. 0.8 µg/g; *p* < 0.001) and delivered at younger ages (median 25.8 vs. 29.4 years; *p* = 0.001). In addition, interior women were more often less educated (85.0% vs. 20.8%; *p* < 0.001) and had lower household incomes (88.7% vs. 32.2%; *p* < 0.001). The timing of the first ANC visit was later in pregnancy for interior participants (median 16 vs. 8 weeks of pregnancy; *p* < 0.001). We analyze the sociodemographic variables for each separate geographic region and did not find any significant association with the subset Paramaribo. Compared with Paramaribo, in the interior subset we found low Hg levels, young maternal age to be associated with ABO/PTB for the Indigenous participants (see Supplementary Table S4).

**Figure 2:**
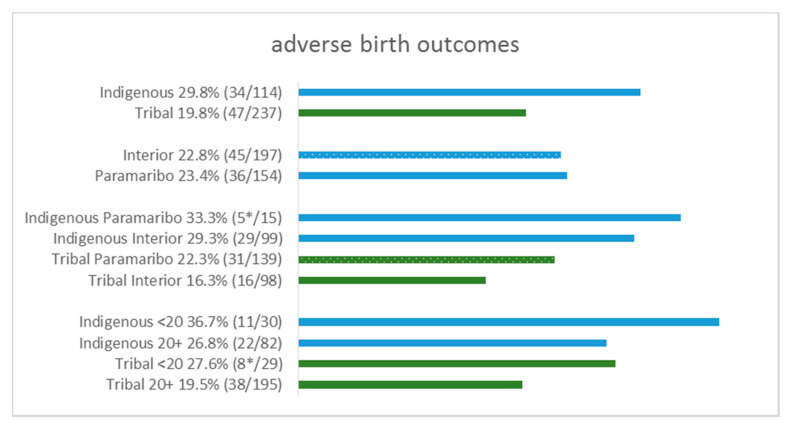
Rates of adverse birth outcomes stratified for ethnic background, geographical background, and age at delivery. * Rates are based on fewer than 10 ABO. Two-sample test for differences in proportions: Indigenous vs. Tribal (*p* = 0.043); Interior vs. Paramaribo (*p* = 1.000); Indigenous Interior vs. Indigenous Paramaribo (*p* = 0.767); Tribal Interior vs. Tribal Paramaribo (*p* = 0.321); Indigenous 16–19 years vs. Indigenous 20+ years (*p* = 0.353); Tribal 16–19 years vs. Tribal 20+ years (*p* = 0.328).

**Figure 3:**
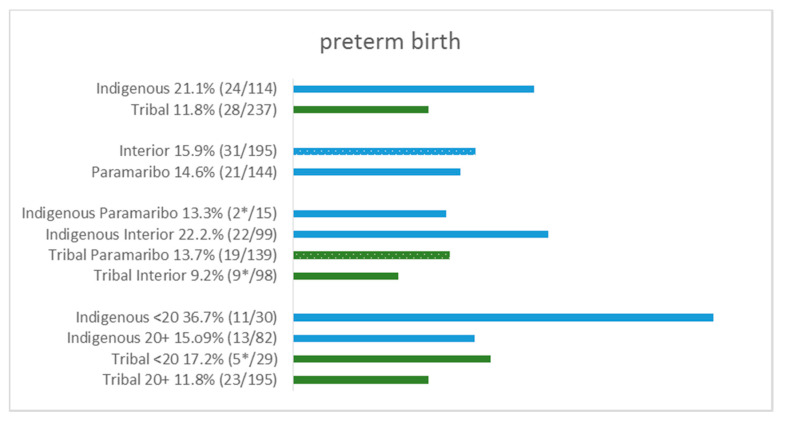
Rates of preterm birth stratified for ethnic background, geographical background, and age at delivery. * Rates are based on fewer than 10 PTB. Two-sample test for differences in proportions: Indigenous vs. Tribal (*p* = 0.025); Interior vs. Paramaribo (*p* = 0.763); Indigenous Interior vs. Indigenous Paramaribo (*p* = 0.734); Tribal Interior vs. Tribal Paramaribo (*p* = 0.316); Indigenous 16–19 years vs. Indigenous 20+ years (*p* = 0.035); Tribal 16–19 years vs. Tribal 20+ years (*p* = 0.377).

The overall rate of ABO was 23.1% (5.4% stillbirths, 15.3% PTB, 9.4% LBW, 3.1 low Apgar score). Rates of ABO (29.8% vs. 19.8%; Figure 2) and PTB (21.2% vs. 12.4%; Figure 3) were significantly higher among Indigenous participants than Tribal participants. Stratification by geographical background and maternal age revealed that ABO were highest among Indigenous participants residing in Paramaribo (33.3%) and among Indigenous participants aged 16–19 years (36.7%), while Tribal participants in the interior had the lowest proportions (16.3%). Highest rates of ABO were observed for Indigenous and Tribal participants aged 16–19 years (36.7% and 27.6%, respectively). Median Hg levels were lower among participants with ABO (Supplementary Table S1); differences in the distribution were not significant except for the Apgar score (median 1.4 (0.6–2.4) for normal vs. 2.1 (0.9–6.2) for low Apgar score; *p* = 0.036). Restricting to interior population only did not reveal significant differences in distribution of Hg levels although observed median Hg levels were high among participants with vs. without ABO and among participants with vs. without PTB (Supplementary Table S2).

Compared with Tribal participants, Indigenous participants had higher proportions of overall ABO (29.8% vs. 19.8%; *p* = 0.043; Figure 2) and PTB (21.2% vs. 12.4%; *p* = 0.025; Figure 3). Comparison by region revealed that interior participants had lower proportions of stillbirths compared to those from Paramaribo (3.0% vs. 9.7%; *p* = 0.012); rates for ABO were also higher for participants without previous live births than participants with previous live births (32.1% vs. 20.5%; *p* = 0.031) (Table 3). Participants with PTB were significantly younger (median 24.0 vs. 27.8; *p* = 0.016) than those with term births.

Multivariate logistic regression analysis (Table 4) revealed that Indigenous participants had higher odds of ABO compared with Tribal participants (adjusted OR = 3.6; 95% CI 1.70–7.63; *p* = 0.001) and lower odds of ABO for increased Hg exposure during pregnancy (OR = 0.88; 95% CI 0.80–0.97; *p* = 0.011). Indigenous participants had higher odds of PTB compared with Tribal participants (adjusted OR = 3.4; 95% CI 1.48–7.96; *p* = 0.004) and lower odds of PTB for increased Hg exposure (OR = 0.88; 95% CI 0.88–0.99; *p* = 0.027), as well as for maternal age at delivery (Table 4). Excluding ethnic background Indigenous and Tribal participants from the multivariate logistic regression model revealed no association between other maternal sociodemographics and ABO, while for PTB did an association with maternal age (p=0.004) was found (Supplementary Table S3).

## 4. Discussion

We examined the association between maternal Hg exposure and social determinants on birth outcomes of pregnant Indigenous and Tribal women in Suriname. Compared with Tribal participants, the Indigenous participants had significantly higher proportions of ABO, especially PTB. Hg exposure was significantly higher among Indigenous participants. Additionally, Indigenous participants were significantly less educated and had significantly lower household incomes. ABO and social determinants did not differ for the Indigenous population living in Paramaribo compared to those in the interior, except that stillbirths were significantly lower in the interior.

ABO prevalence was high among the Indigenous population compared to the Tribal population. Evidence from 16 low- and middle-income countries across the globe indicates that Indigenous women experience significantly worse maternal health outcomes than the non-indigenous population [27]. Earlier research on ethnic disparities and childbirth in Suriname has shown that infants born to Tribal mothers have a high risk for ABO such as stillbirth, PTB, and low Apgar score, compared to other ethnicities (Hindustani, Creole, Javanese, Chinese, Indigenous, and mixed) in Suriname [1]. Our findings of higher ABO among Indigenous women compared to Tribal women could be explained by the origins of the Indigenous population in Suriname. These Indigenous tribes live in very remote parts of the interior, and their communities are only reachable by airplane or by boat after days of traveling, with less access to ANC including skilled birth attendants [28,29]. The Indigenous women were also younger than the Tribal women and started later with ANC visits. Having a child before 18 years (early childbearing) is documented as occurring in Native American women as well [30]. There are many factors associated with early childbearing risk that might apply to Indigenous women in Suriname, such as ethnic minority status, being a daughter of a teenage mother, lower socioeconomic status, residence in remote and rural areas, and less access to comprehensive sex education or lack of access to and use of contraceptives [31,32,33,34]. Pregnancy-related biological factors such as maternal blood pressure, maternal weight, pregnancy-induced hypertension, preeclampsia, and infections (urine tract infections, malaria) are other factors that could lead to an increased risk of ABO [35,36]. In this study, we only included a few biological factors, such as age and parity, as the focus was on sociodemographic determinants; however, including these factors will be a next step in this research to explore these associations more thoroughly. The health systems analysis also brought to light differences in the types and comprehensive inclusion of biological markers between the interior and the Paramaribo region. Specifically, Paramaribo’s prenatal visits included a more comprehensive set of biological markers. These regional differences precluded the inclusion of a more comprehensive set of biological markers in this study.

Overall, PTB was higher in the Indigenous population compared to the Tribal population. Our study also revealed increased odds of PTB neonates for Indigenous births in the capital but not in the interior of Suriname. This might be explained by the secluded lifestyle of this ethnic group in the capital, as they are often hesitant to get access to care due to fear of discrimination, lack of finances, and language barriers [27]. A large systematic review and meta-analysis done in the US concluded that women living in the most disadvantaged geographic areas have a significantly higher risk for PTB (RR = 1.27; 95% CI: 1.16–1.39) compared to those in the least disadvantaged areas, but the association may differ by ethnicity [37]. Additionally, a meta-analysis study done in China revealed that maternal BMI is associated with adverse birth outcomes; maternal overweight is associated with PTB and maternal underweight with LBW and being small for gestational age [38]. This is in contrast other studies conducted in low- and middle-income countries, which revealed that women who were underweight had a higher risk for PTB [39,40]. Therefore, more research is needed to verify our findings.

The World Health Organization recommends that pregnant women living in developing countries should start antenatal care before 12 weeks of pregnancy for better birth outcomes [41]. In our study, although all women came for ANC visits, the number of visits was not recorded, and more than half of all participants began ANC visits after 12 weeks (60%). Late initiation of ANC was more evident in the Indigenous study population compared to the Tribal group. In our study, women from the interior had later ANC initiation compared to those in Paramaribo, and they were also significantly younger in age. Indigenous women are generally less likely to seek early ANC; however, little is known as to why [27,42]. We assume that, since women in the Indigenous population were younger in our study population, they could have started later with ANC because of unintended pregnancies (e.g., little or no access to contraception, cultural beliefs, or institutional barriers, such as a long wait time for an appointments and not trusting the healthcare provider, all of which have been associated with late ANC initiation in Native American women) [43]. It is therefore necessary to have tailored awareness programs for the Indigenous population that may stimulate seeking earlier access to health care to positively impact both social and psychological well-being. Incorporation of intercultural approaches to sexual and reproductive health (SRH) services and focusing on school SRH educational programs should be prioritized by the Surinamese government for the Indigenous population to reduce the adolescent birth rate within this ethnic group, as 16.8% of the study population delivered before <20 years, which is higher than the country-wide statistics (13.8%), although it is somewhat lower than Colombia (17%), Guyana (19–22%), and Haiti (27%) [1,44,45].

This study did not find an association between Hg exposure and ABO. Hg exposure was higher in the interior and among the Indigenous study population. After stepwise regression, we found Hg exposure to be associated with PTB. These findings are similar to other studies on Hg and reproductive outcomes, although not all studies show consistent results [46,47]. High concentrations of Hg have been found in fish from the interior of Suriname [24,25] Indigenous populations in the interior highly depend on fish consumption for their source of daily protein. Our study revealed that lower Hg exposure was associated with increased odds for ABO and PTB. High fish consumption may have protective effects, since these freshwater fish are rich in selenium, zinc, polyunsaturated fatty acids, and other important micronutrients, which could potentially counteract the effect of Hg exposure [46,48,49] Although fish provides a highly nutritious food source, the health impacts of Hg exposure, such as neurobehavioral deficits, cardiovascular toxicity, and immunological effects, have been reported both for adults and children in several Amazonian countries [20,50]. Long-term cognitive impacts of mercury exposure were not evaluated in children. The ongoing CCREOH cohort study will follow the children born to these exposed mothers to assess any neurodevelopmental effects. Meanwhile, a nutritionally balanced diet including appropriate fish selection and consumption guidance should be recommended for the Indigenous populations to reduce the potential risk of adverse health effects due to Hg exposure. Future research and epidemiology studies with larger samples are needed to verify these findings.

Young maternal age was associated with PTB for Indigenous participants from the interior. In line with previous studies, increased risk of ABO including PTB was associated with adolescent maternity [51,52,53,54]. In some studies, this association did not hold after controlling for socioeconomic and reproductive factors, thus indicating that social disadvantage rather than biological factors may be the explanation [6]. However, in other studies, the association persisted after adjustment. In Brazil, researchers found the relationship between young maternal age and PTB may either have a biological origin or result from errors in gestational age estimation [55]. We ran the analysis after excluding adolescent pregnancies (<20 years) and found no association between maternal age and ABO (*p* = 0.131) or PTB (*p* = 0.359). Health care services in the interior of Suriname do not provide ultrasound examination as part of their regular prenatal care portfolio; thus, the gestational age is calculated with the last menstrual period (LMP) of the women. This method may not accurately estimate gestational age as cycle characteristics and the date of onset of the last menstrual bleed must be clearly established, yet this has proven difficult in many low- and middle-income settings [56]. There were higher odds of PTB among nulliparous mothers, adjusting for education, maternal age, and region. These findings are similar to other studies [57,58]. Other maternal characteristics have been associated with PTB such as lower or high body mass index (BMI) or smoking [58,59,60]. Health care providers need to take these risk factors into consideration as they strive to collect information on maternal obstetric history to indicate the risk of PTB.

Although education level and household income were significantly lower for Indigenous participants in the interior (*p* < 0.001), we did not find any association with ABO. In contrast, a study conducted in North Ethiopia found factors such as maternal education, residence, husband’s education, and monthly household income to be significantly associated with ABO [61]. A study conducted in the US observed an influence of maternal education on PTB and found relative risks ranging from 1.28 to 1.67 for PTB, with Black women with less education having a 1.67 times higher risk of PTB [62]. Disparity in PTB rates has been found in populations where women of different races are afforded the same access to healthcare services, although the results are not consistent [63,64]. It is therefore likely that rates of PTB were further influenced by other risk factors such as interpregnancy interval, psychosocial stress, and genetic variation, rather than those that were taken into account in the current study; many other preterm births are unexplained merely by sociodemographic differences as they are interrelated [63]. Furthermore, the differences observed between Tribal and Indigenous women were more apparent in the interior because of the difference in cultural lifestyle of these two groups, who do not mingle. In addition, the Indigenous population lives in more isolated areas only accessible by air, and thus, has less contact with the outside world. In addition, some of these Indigenous villages lack adequate education since there are no schools.

Strengths of our study include the prospective study design and the inclusion of a unique population that has not been well-studied for risks for ABO. Additionally, this study is the first to compare ABO of Indigenous and Tribal women in Suriname. Some limitations include the percentage (5%) of missing data for birth outcomes; however, neo- and perinatal data for the Indigenous and Tribal women living in the interior are not always available or well documented in Suriname. This is an important medical care data gap that needs more attention. Second, there are differences in pre- and perinatal care delivery between Paramaribo and the remote interior. For example, regular ultrasound examination during pregnancy in Paramaribo is common, while there is essentially no ultrasound examination in the interior. This may explain why the interior had high estimates of PTB as the gestational age in the interior was calculated with LMP and may well be underestimated. Third, important causes of PTB, such as genitourinary infection, multiple pregnancies, pregnancy-induced hypertension, low pre-pregnancy BMI, incompetent cervix, history of prior preterm birth, and cigarette smoking, were not analyzed in this study. Fourth, as stated previously, our current study could not fully explain differences in birth outcomes between the two populations in the different regions since multiple biological determinants were not included in the analyses. Including these will be an important step in our ongoing research trajectory. Finally, we acknowledge that the sample size of the Indigenous group in Paramaribo was relatively small for adequate comparison and data analyses. The imbalance between the Tribal and Indigenous groups would seem to make comparisons somewhat contingent on the fact that more women in the interior are Indigenous and exposed to more Hg, while more women in Paramaribo are Tribal and exposed to less Hg.

## 5. Conclusions

In conclusion, Indigenous participants had higher odds of ABO compared with Tribal participants, independent of their parity and Hg exposure during pregnancy. Ethnic background, maternal age, and Hg exposure during pregnancy were independent predictors of PTB, which is in line with other studies. Indigenous participants had higher odds of PTB compared to Tribal participants. This study is novel for Suriname, and these results highlight the importance of identifying and implementing risk reduction measures that are effective and in support of Indigenous women, families, and communities. Differences in prenatal care delivery services by regions are evident, for example, interior participants do not have access to ultrasound and full blood count tests. Such empirical evidence could be useful for the development of tailored health policies for Indigenous women in Suriname, as well as the knowledge of community members and other stakeholders to better inform risk reduction policies. Differences in healthcare services and ANC delivery provided in the interior and Paramaribo could be explanatory factors for these results rather than sociodemographic or biological factors; therefore, research on accessibility to healthcare is necessary to further develop strategies for prenatal care. We further recommend improving the dissemination of essential information about ANC through trusted healthcare providers and community networks to ensure that pregnant Indigenous women maximally benefit from these services, especially those living in the interior. Programs should ideally be holistic, affect multiple scales (individual, family, and community), and be adequately supported. Furthermore, Suriname as a member country of the Minamata Convention should promote mercury-free gold-processing methods; take special measures to protect vulnerable populations, including children and women of childbearing age from exposure; and put an end to particularly harmful practices in gold processing.

## Figures and Tables

**Table 1:** Characteristics of the Indigenous and Tribal study participants.

Variables	Indigenous (*n* = 114)		Tribal (*n* = 237)		*p*-Value
	*n*	%	*n*	%	
Hair Hg					
Median [IQR]	105	7.9 [3.7–11.0]	199	1.4 [0.7–2.3]	<0.001
Hg exposure					
Low + medium	24	22.9%	179	89.9%	<0.001
High	81	77.1%	20	10.1%	
Age					
Median [IQR]	114	25.1 [20.0–30.7]	237	29.1 [22.5–34.5]	0.001
Age					
16–19	31	27.2%	32	13.5%	0.002
20–34	70	61.4%	156	65.8%	
35+	13	11.4%	49	20.7%	
Parity					
no previous live births	30	26.3%	51	21.7%	0.338
1+ previous live births	84	73.7%	184	78.3%	
Educational level					
primary or not	90	79.6%	106	45.3%	<0.001
secondary and up	23	20.4%	128	54.7%	
Household income in SRD					
<1500	94	83.9%	117	53.9%	<0.001
1500+	18	16.1%	100	46.1%	
Timing of first antenatal visit					
Median [IQR]	111	15 [9–21]	224	12 [7–16]	0.002
Timing of first antenatal visit					
<12 weeks	34	30.6%	99	44.2%	0.017
12+ weeks	77	69.4%	125	55.8%	

Associations between categorical variables were tested with the Pearson X-test. Differences in median values were tested with the Mann–Whitney U-test.

**Table 2:** Characteristics of participants stratified for geographic region.

Variables	Interior (*n* = 197)		Paramaribo (*n* = 154)		*p*-Value
	*n*	%	*n*	%	
Hair Hg					
Median [IQR]	185	3.6 [2.1–8.7]	119	0.8 [0.4–1.4]	<0.001
Hg exposure					
Low + medium	87	47.0%	116	97.5%	<0.001
High	98	53.0%	3	2.5%	
Age					
Median [IQR]	197	25.8 [20.5–32.0]	154	29.4 [23.8–34.9]	0.001
Age					
16–19	45	22.8%	18	11.7%	0.010
20–34	124	62.9%	102	66.2%	
35+	28	14.2%	34	22.1%	
Parity					
no previous live births	39	20.0%	42	27.3%	0.110
1+ previous live births	156	80.0%	112	72.7%	
Ethnic background					
Indigenous	99	50.3%	15	9.7%	<0.001
Tribal	98	49.7%	139	90.3%	
Educational level					
primary or not	164	85.0%	32	20.8%	<0.001
secondary and up	29	15.0%	122	79.2%	
Household income in SRD					
<1500	165	88.7%	46	32.2%	<0.001
1500+	21	11.3%	97	67.8%	
Timing of first antenatal visit					
Median [IQR]	187	16 [12–21]	148	8 [5–14]	<0.001
Timing of first antenatal visit					
<12 weeks	35	18.7%	98	66.2%	<0.001
12+ weeks	152	81.3%	50	33.8%	

Differences in median values by ethnic subgroups were tested with the Mann–Whitney U-test. Differences in proportions by ethnic subgroups were tested with the two-sample test of proportion.

**Table 3:** Characteristics of participants stratified for adverse birth outcomes and for preterm birth.

Variables	ABO Yes (*n* = 81)		ABO No (*n* = 270)		*p*-Value	PTB Yes (*n* = 52)		PTB No (*n* = 287)		*p*-Value
Ethnic background										
Indigenous	34	29.8%	80	70.2%	**0.037**	24	21.2%	89	78.8%	0.033
Tribal	47	19.8%	190	80.2%		28	12.4%	198	87.6%	
Geographic area										
Interior	45	22.8%	152	77.2%	0.906	31	15.9%	164	84.1%	0.740
Paramaribo	36	23.4%	118	76.6%		21	14.6%	123	85.4%	
Hair Hg										
Median [IQR]	69	1.7 [0.7–6.5]	235	2.1 [1.0–5.8]	0.460	47	1.8 [0.7–6.8]	250	2.1 [1.0–5.8]	0.454
Hg exposure										
Low + medium	46	22.7%	157	77.3%	0.982	30	15.3%	166	84.7%	0.733
high	23	22.8%	78	77.2%		17	16.8%	84	83.2%	
Age										
Median [IQR]	81	27.0 [19.8–33.8]	270	27.7 [22.2–32.9]	0.385	52	24.0 [19.1–31.3]	287	27.8 [22.2–33.2]	0.016
Age										
16–19	21	33.3%	42	66.7%	0.080	16	25.8%	46	74.2%	0.034
20–34	45	19.9%	181	80.1%		30	13.6%	190	86.4%	
35+	15	24.2%	47	75.8%		6	10.5%	51	89.5%	
Parity										
no previous live births	26	32.1%	55	67.9%	0.031	14	18.7%	61	81.3%	0.379
1+ previous live births	55	20.5%	213	79.5%		38	14.5%	224	85.5%	
Educational level										
primary or not	41	20.9%	155	79.1%	0.428	27	14.1%	165	85.9%	0.735
secondary and up	37	24.5%	114	75.5%		22	15.4%	121	84.6%	
Household income in SRD										
<1500	45	21.3%	166	78.7%	0.396	28	13.5%	179	86.5%	0.092
1500+	30	25.4%	88	74.6%		19	17.0%	93	83.0%	
Timing of first antenatal visit										
Median [IQR]	113	12.5 [5.75–18]	246	13 [8.5–18]	0.103	47	12 [5–18]	276	13.5 [9–18]	0.227
Timing of first antenatal visit										
<12 weeks	33	24.8%	100	75.2%	0.330	22	17.7%	102	82.3%	0.199
12+ weeks	41	20.3%	161	79.7%		25	12.6%	174	87.4%	

Differences in median values by ethnic subgroups were tested with the Mann–Whitney U-test. Differences in proportions by ethnic subgroups were tested with the two-sample test of proportion.

**Table 4:** Crude and adjusted odds ratios (OR) with 95% confidence intervals (95% CI) for adverse birth outcomes and for preterm birth.

Adverse Birth Outcomes (Yes vs. No)					Outcome Preterm Birth (Yes vs. No)				
**Crude model**	***p*-Value**	**Crude OR**	**95% CI**		**Crude model**	***p*-Value**	**Crude OR**	**95% CI**	
			LB	UB				LB	UB
Indigenous vs. Tribal	0.039	1.72	1.03	2.87	Indigenous vs. Tribal	0.035	1.91	1.05	3.47
**Adjusted model with all covariates**	***p*-value**	**Adjusted OR**	**95% CI**		**Adjusted model with all covariates**	***p*-value**	**Adjusted OR**	**95% CI**	
			LB	UB				LB	UB
Indigenous vs. Tribal	0.007	3.11	1.36	7.08	Indigenous vs. Tribal	0.017	3.23	1.24	8.44
Paramaribo vs. Interior	0.820	1.12	0.44	2.86	Paramaribo vs. Interior	0.366	1.67	0.55	5.07
Age in years	0.517	0.99	0.94	1.03	Age in years	0.004	0.92	0.87	0.97
Parity no vs. 1+ previous live births	0.319	1.44	0.70	2.97	Parity no vs. 1+ previous live births	0.220	0.58	0.24	1.39
Education primary or not vs. secondary and up	0.649	0.82	0.35	1.92	Education primary or not vs. other	0.793	0.88	0.33	2.35
Household income <1500 vs. other	0.767	0.88	0.39	2.02	Household income <1500 vs. other	0.414	0.67	0.25	1.76
First antenatal visit <12 vs. 12+ weeks	0.541	0.81	0.41	1.61	First antenatal visit <12 vs. 12+ weeks	0.533	0.78	0.36	1.71
Hg exposure	0.119	0.92	0.83	1.02	Hg exposure	0.357	0.95	0.84	1.06
**Final adjusted model (stepwise procedure)**	***p*-value**	**Adjusted OR**	**95% CI**		**Final adjusted model (stepwise procedure)**	***p*-value**	**Adjusted OR**	**95% CI**	
			LB	UB				LB	UB
Indigenous vs. Tribal	0.001	3.60	1.70	7.63	Indigenous vs. Tribal	0.004	3.43	1.48	7.96
Hg exposure	0.011	0.88	0.80	0.97	Hg exposure	0.027	0.88	0.79	0.99
					Age in years	0.040	0.95	0.91	1.00

## Data Availability

The data presented in this study can be made available based on a reasonable request. Such requests should be directed to the PI’s of the Caribbean Consortium for Research in environmental and Occupational Health (CCREOH) through the intermediary of the corresponding author.

## Supplementary Materials

The following are available online at https://www.mdpi.com/article/10.3390/ijerph18126370/s1, Table S1: Mercury levels and adverse birth outcomes; Table S2: mercury levels and adverse birth outcomes for Interior population; Table S3: Adjusted Odds ratio (OR) with 95% Confidence Interval (95% CI) for adverse birth outcomes and for preterm birth without ethnicity; Table S4: Characteristics of the study population by geographic region.
