# Supplementary material for: Association of Mercury Exposure and Maternal Sociodemographics on Birth Outcomes of Indigenous and Tribal Women in Suriname
Source: Int J Environ Res Public Health. 2021 Jun 12;18(12):6370. doi: 10.3390/ijerph18126370 (PMC8296187; doi:10.3390/ijerph18126370)
Supplement: Supplementary file 1 [file ijerph-18-06370-s001.zip › ijerph-1216576-supplementary.pdf]

International Journal of Environmental Research and Public Health

Table S1. Mercury levels and adverse birth outcome.

Interior and Paramaribo					
**ABO**	**Yes**		**No**		
Hair Hg	*n*	Median [IQR]	*n*	Median [IQR]	*p*-value
ABO	69	1.7 [0.7–6.5]	235	2.1 [1.0–5.8]	0.460
SB	15	1.6 [0.7–-2.8]	289	2.1 [0.9–6.0]	0.197
PTB	47	1.8 [0.7–6.8]	250	2.1 [1.0–5.8]	0.454
LBW	30	1.4 [0.7–3.1]	263	2.2 [1.0–6.2]	0.085
LAS	20	1.4 [0.6–-2.4]	284	2.1 [0.9–6.2]	0.036

Table S2. Mercury levels and adverse birth outcome for Interior population.

Interior					
**ABO**	**Yes**		**No**		
Hair Hg	*n*	Median [IQR]	*n*	Median [IQR]	*p*-value
ABO	41	4.4 [1.8–9.2]	144	3.5 [2.1–8.6]	0.802
Stillbirth	6	3.3 [1.5–5.6]	179	3.6 [2.1–8.8]	0.448
PTB	27	6.5 [2.1–9.2]	156	3.5 [2.1–8.8]	0.532
LBW	18	2.2 [1.4–7.9]	164	3.7 [2.1–8.8]	0.096
Low Apgar Score	7	2.8 [0.9–4.4]	178	3.6 [2.1–8.9]	0.174

Table S3. Adjusted Odds ratio (OR) with 95% Confidence Intervals (95% CI) for adverse birth outcomes and for preterm birth without ethnicity.

Adverse Birth Outcomes (yes vs. no)					Outcome Preterm Birth (yes vs. no)				
**Adjusted model**	***p*-value**	**Crude OR**	**95% CI**		**Adjusted model**	***p*-value**	**Crude OR**	**95% CI**	
			**LB**	**UB**				**LB**	**UB**
Paramaribo vs. Interior	0.577	0.777	0.32	1.885	Paramaribo vs. Interior	0.924	1.051	0.379	2.912
Age in years	0.442	0.982	0.939	1.028	Age in years	0.004	0.919	0.868	0.973
Parity no vs. 1+ previous live births	0.279	1.482	0.727	3.023	Parity no vs. 1+ previous live births	0.275	0.618	0.261	1.466
Education primary or not vs. else	0.509	0.754	0.326	1.744	Education primary or not vs. else	0.659	0.803	0.304	2.126
Household income <1500 vs. else	0.667	0.837	0.373	1.881	Household income <1500 vs. else	0.342	0.63	0.243	1.633
First antenatal visit <12 vs. 12+ weeks	0.34	0.721	0.367	1.413	First antenatal visit <12 vs. 12+ weeks	0.337	0.685	0.316	1.482
Hg exposure	0.846	0.992	0.916	1.075	Hg exposure	0.74	1.015	0.928	1.11

Table S4. Characteristics of the study population by geographic region.

Variables	Paramaribo					Interior				
	Indigenous (*n*)	%	Tribal(*n*)	%	*p*-value	Indigenous (*n*)	%	Tribal(N)	%	*p*-value
ABO	5	33.30	31	22.30	0.338	29	29.30	16	16.30	**0.030**
Stillbirth	2	13.30	11	7.90	0.473	2	2.00	4	4.10	0.400
PTB	2	14.30	19	14.60	0.974	22	22.20	9	9.40	**0.014**
LBW	3	21.40	12	9.40	0.164	8	8.20	10	10.40	0.589
Low Apgar score	2	14.30	6	4.70	0.139	2	2.00	5	5.10	0.243
Hg exposure										
lowmedium	8	88.90	108	98.20	0.087	16	16.70	71	79.80	**<0.001**
high	1	11.10	2	1.80		80	83.30	18	20.20	
Age										
16-19	2	13.30	16	11.50	0.863	29	29.30	16	16.30	**0.025**
20-34	9	60.00	93	66.90		61	61.60	63	64.30	
35+	4	26.70	30	21.60		9	9.10	19	19.40	
Parity										
no previous live births	5	33.30	37	26.60	0.579	25	25.30	14	14.60	0.063
1+ previous live births	10	66.70	102	73.40		74	74.70	82	85.40	
Educational level										
primary or not	4	26.70	28	20.10	0.554	86	87.80	78	82.10	0.272
secondary and up	11	73.30	111	79.90		12	12.20	17	17.90	

Household income in SRD										
<1500	3	21.40	43	33.30	0.365	91	92.90	74	84.10	0.059
1500+	11	78.60	86	66.70		7	7.10	14	15.90	
Timing of first antenatal visit										
<12 weeks	11	73.30	87	65.40	0.539	23	24.00	12	13.20	0.059
12+ weeks	4	26.70	46	34.60		73	76.00	79	86.80	
